# Mechanisms of Resistance, Insights From Case Reports, and Future Prospects in Mycobacteriophage Therapy

**DOI:** 10.1002/snz2.70064

**Published:** 2026-07-11

**Authors:** Buhari Yusuf, Abdul Malik, Md Shah Alam, Lijie Li, Haftay Abraha Tadesse, Aweke Mulu Belachew, Cuiting Fang, Xirong Tian, Htin Lin Aung, Shuai Wang, Tianyu Zhang

**Affiliations:** ^1^ State Key Laboratory of Respiratory Disease Guangzhou Institutes of Biomedicine and Health Chinese Academy of Sciences Guangzhou China; ^2^ Guangdong‐Hong Kong‐Macao Joint Laboratory of Respiratory Infectious Diseases Guangzhou Institutes of Biomedicine and Health Chinese Academy of Sciences Guangzhou China; ^3^ University of Chinese Academy of Sciences Beijing China; ^4^ School of Life Sciences University of Science and Technology of China Hefei China; ^5^ Guangzhou Medical University‐Guangzhou Institutes of Biomedicine and Health Joint School of Life Sciences Guangzhou Medical University Guangzhou China; ^6^ State Key Laboratory of Respiratory Disease Department of Tuberculosis Guangzhou Chest Hospital Institute of Tuberculosis Guangzhou Medical University Guangzhou China; ^7^ Department of Microbiology and Immunology School of Biomedical Sciences University of Otago Dunedin New Zealand; ^8^ Maurice Wilkins Centre for Molecular Biodiscovery University of Auckland Auckland New Zealand; ^9^ Guangzhou National Laboratory Guangzhou China

**Keywords:** mycobacteria, mycobacterial infections, mycobacteriophage therapy, mycobacteriophages, nontuberculous mycobacteria

## Abstract

Evolution of drug‐resistant mycobacterial infections warrants renewed efforts in identifying more efficient preventive and control strategies as well as alternative treatment options. Interest in phage therapy is regaining significant traction, especially in cases of failed conventional therapy. However, phage therapy faces challenges, including the identification of a suitable therapeutic phage, phage delivery, phage resistance, and host immunity. This article reviews existing clinical literature on the therapeutic use of mycobacteriophages as adjuncts to antibiotics in the treatment of drug‐resistant mycobacterial infections and discusses such aspects as mycobacterial phage resistance, coevolutionary phage training, and impact of host immunity, as well as the benefits and limitations of phage therapy. To date, at least 27 patients received mycobacteriophage therapy, where *M. abscessus* accounts for 82.1% of all cases in contrast to *M. chelonae* (7.1%), *M. avium* complex (3.6%), and BCG (3.6%). Mycobacteriophages used were either wild‐type or derivatives of BPs, D29, Fionnbharth, Fred313, Itos, Muddy, or ZoeJ. Evolution of phage resistance is rare, and the impact of host immunity varies between patients, with most treatment outcomes having little to do with immune responses. However, identification of a suitable therapeutic mycobacteriophage remains a pressing challenge, especially for infections involving the smooth morphotype of *M. abscessus*. Only about 10 mycobacteriophages made it to clinical use, including wild‐type, host range mutants and engineered derivatives, which warrants the expansion of this narrow arsenal of therapeutic mycobacteriophages by building novel candidates or expanding the host ranges of existing ones. The outcomes recorded in these case reports represent a significant achievement. However, the results remain exploratory due to sample size limitations and therefore warrant larger, methodologically rigorous, controlled trials in the future.

## 1 Introduction

The discovery of antibiotics is one of the great turning points in the history of modern medicine, which came at a time when the slightest of infections could be fatal (Cooper and Shlaes 2011; Tommasi et al. 2015). Beginning in the first half of the 20^th^ century, antibiotic therapy has transformed the management and control of infectious diseases and therefore improved healthcare delivery. However, the rate at which bacteria evolve exceeds the rate at which new antibiotics are developed. Drug resistance genes are spread among bacteria in clinical, agricultural, and natural environments, which reflects the danger associated with inappropriate/unchecked use of antibiotics, often leading to evolution of broad‐spectrum antimicrobial resistance (AMR) and untreatable infections (Levy and Marshall 2004; Davies and Davies 2010; Zhang et al. 2009; Unemo and Shafer 2014). As a consequence, antibiotics are increasingly becoming less effective with the evolution of antimicrobial‐resistant bacteria, hence the need for alternative therapy to current antibiotics.

TB holds the distinction of being the inaugural infectious disease to be designated as a global health emergency, and over the past three decades, it has become a leading cause of death due to AMR. Though not officially recognized by WHO, the term “totally drug‐resistant TB” has been used in multiple reports to describe TB resistance to all tested anti‐TB drugs (Migliori et al. 2007; Migliori et al. 2007; Migliori et al. 2007; Velayati et al. 2009; Udwadia et al. 2012; Malathy 2012). It is estimated that with the current trends, mortalities due to antibiotic‐resistant bacterial infections could reach over 39 million between 2025 and 2050 (GBD 2021).

Phages (or bacteriophages) are bacteria‐eating viruses that can infect and kill bacteria without causing harm to the human or animal host. Phages encode special cell wall hydrolases called lysins (or endolysins), which degrade bacterial peptidoglycan, destroy cells, and release progeny viruses. Because of their diversity in origin, evolution, and genome organization, phages are highly specific and therefore present a good strategy to replace prolonged and toxic antibiotic therapy in an approach that is far more specific than antibiotics (Hatfull 2014; Ajuebor et al. 2016).

Phage therapy is the use of bacteriophages to treat bacterial infections (Kortright et al. 2019; Schooley and Strathdee 2020; Schooley et al. 2017; Dedrick et al. 2019; Aslam et al. 2019). Endolysins are also considered as potentially effective therapeutic candidates against bacterial infections (Loessner 2005; Nelson et al. 2001). This concept has been around for over a century but renewed interest in its utility as an alternative to antibiotic therapy has been due to the rising AMR crisis. Phage therapy has demonstrated its potential as a last‐resort treatment for life‐threatening multidrug‐resistant bacterial infections (Schooley et al. 2017; Dedrick et al. 2019; Aslam et al. 2019; Torres‐Barceló 2018; Chan et al. 2018) and is lately reconsidered as a way of treating bacterial infections globally.

## 2 Phage–Host Interaction

**FIGURE 1:**
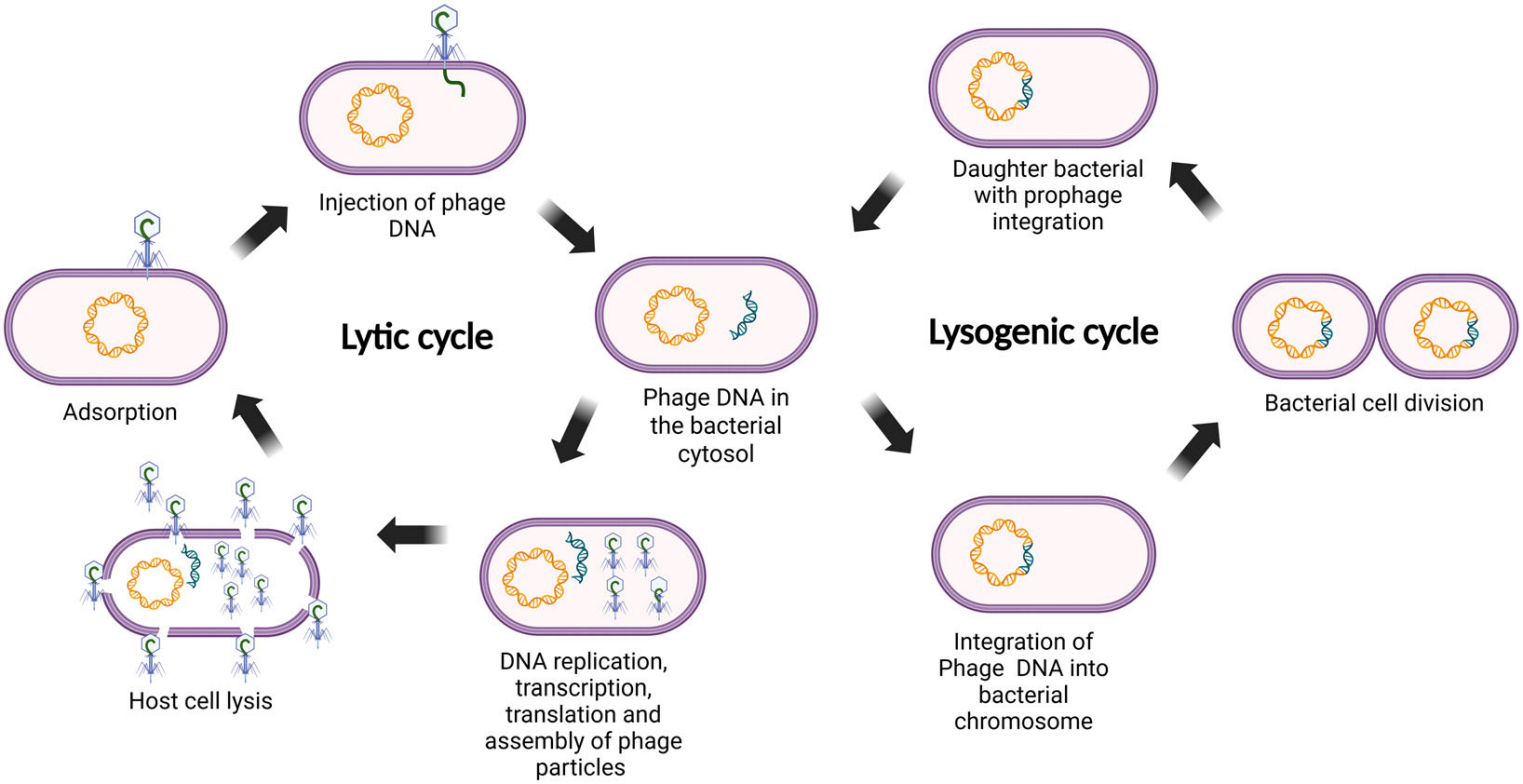
Phage infection cycle. After adsorption and DNA injection, the phage may enter either the lytic or lysogenic cycle depending on its lifestyle. Lytic/virulent phages exclusively follow the lytic path. Temperate phages, however, can choose either of the two or first go through the lysogenic cycle, then enter the lytic lifestyle at a later stage. Exclusively lytic phages are preferred for therapeutic application because they can kill the hosts effectively. Lysogens may become problematic by developing either or both drug and phage resistance. In fact, prophage elements have been detected in clinical isolates of *M. abscessus* (Mageeney et al. 2020), and such elements may confer the isolates with phage resistance (Mageeney et al. 2020; Dedrick et al. 2017; Gentile et al. 2019; Mohammed et al. 2023) and therefore jeopardize the efficacy of phage therapy. Figure was created in BioRender by Malik, A. (2025): https://BioRender.com/jc5gyq5.

Phage infection cycle proceeds in a series of steps, beginning with phage adsorption to the host cell surface usually via specific receptors, followed by DNA injection, DNA replication, transcription and synthesis of viral proteins, assembly of phage particles, and, finally, lysis of the host cell to release progeny viruses (Figure 1). To initiate infection, a phage receptor‐binding protein (RBP) must attach to a specific phage receptor on the bacterial cell surface, and different phages may have different receptor requirements even for the same bacterial host (McKitterick and Bernhardt 2022). These surface receptors could be glycolipids, polysaccharides, or proteins exposed outside or embedded within the cell envelope (Bertozzi Silva et al. 2016; Dunne et al. 2018). In fact, glycolipids are mostly the implicated surface molecules in phage binding to a mycobacterial host (Besra et al. 1994; Bisso et al. 1976; Dhariwal et al. 1986; Furuchi and Tokunaga 1972; Chen et al. 2009; Imaeda and Blas 1969; Wetzel et al. 2023). In *Corynebacterium glutamicum*, which is a related member of the actinobacteria, galactosamine‐modified arabinogalactan, mycolate‐bound trehaloses, and two mycoloylated proteins (ProtX and PorA) have been identified as potential receptors (McKitterick and Bernhardt 2022), further implying the clear significance of sugars and lipids in phage‐host interactions.

Since phage receptors could be components positioned externally or embedded within the bacterial cell wall (McKitterick and Bernhardt 2022), phage‐resistant mutants could be invaluable in determining the biosynthetic pathways for such components of the mycobacterial cell envelope. Inquiries into mechanisms of phage‐host interactions have aided not just the identification of surface receptors, but the understanding of crucial aspects of microbial physiology such as membrane transport, biogenesis of lipids, teichoic acids, and other surface polymers (McKitterick and Bernhardt 2022; Dunne et al. 2018; Heller 1992; Estrela et al. 1991; Tran et al. 1999; Freymond et al. 2006; Randall‐Hazelbauer and Schwartz 1973; Szmelcman and Hofnung 1975; Emr et al. 1978). Because many of these surface molecules are involved in bacterial virulence, dissecting the mechanism of cell envelope assembly also holds the potential to reveal certain vulnerabilities in bacterial metabolism.

Understanding of phage biology has led to the discovery of revolutionary molecular tools for mycobacterial genetics, including nonantibiotic selectable markers (Donnelly‐Wu et al. 1993), plasmid‐shuttle vectors capable of integration (Stover et al. 1991; Haeseleer et al. 1993), transposon delivery systems (Bardarov et al. 1997), reporter phages (Sarkis et al. 1995; Pearson et al. 1996; Jacobs et al. 1993), genome editing tools (van Kessel and Hatfull 2007; Yan et al. 2017), and mycobacterial transfection and transduction (Tokunaga and Sellers 1964; Raj and Ramakrishnan 1970), as well as transformation via the application of shuttle phasmids (Jacobs et al. 1987; Snapper et al. 1988). It is also noteworthy that the discovery of the CRISPR/Cas genome editing system as well as restriction endonucleases also derived inspiration from phage interactions with their hosts.

Another important aspect in the interactions of phages with their hosts is lifestyle, which manifests after injection of phage DNA into the host cell (Figure 1). For lytic phages, downstream processes commence after DNA entry into the bacterial cytosol. For lysogenic phages, however, the injected DNA may remain extrachromosomal or get integrated into the bacterial chromosome, and this particular feature could serve as an exclusion criterion for therapeutic application. This is because the lysogens may harbor phage genes that drive AMR or homotypic/heterotypic phage resistance, both of which are undesirable features for potential therapeutic candidates. However, understanding of phage genomics has now made it possible to generate lytic derivatives from temperate parents for therapeutic applications (Dedrick et al. 2019).

Thus, adequate understanding of phage–host interaction has been invaluable in advancing our knowledge of molecular biology by aiding the development of revolutionary molecular tools, revealing integral aspects of cell envelope assembly in bacteria, and revealing the molecular basis of lysogeny, all of which advance our understanding of phage biology and guide phage engineering efforts for therapeutic applications (Dedrick et al. 2019; Cristinziano et al. 2024).

## 3 Mycobacterial Phage Resistance

**FIGURE 2:**
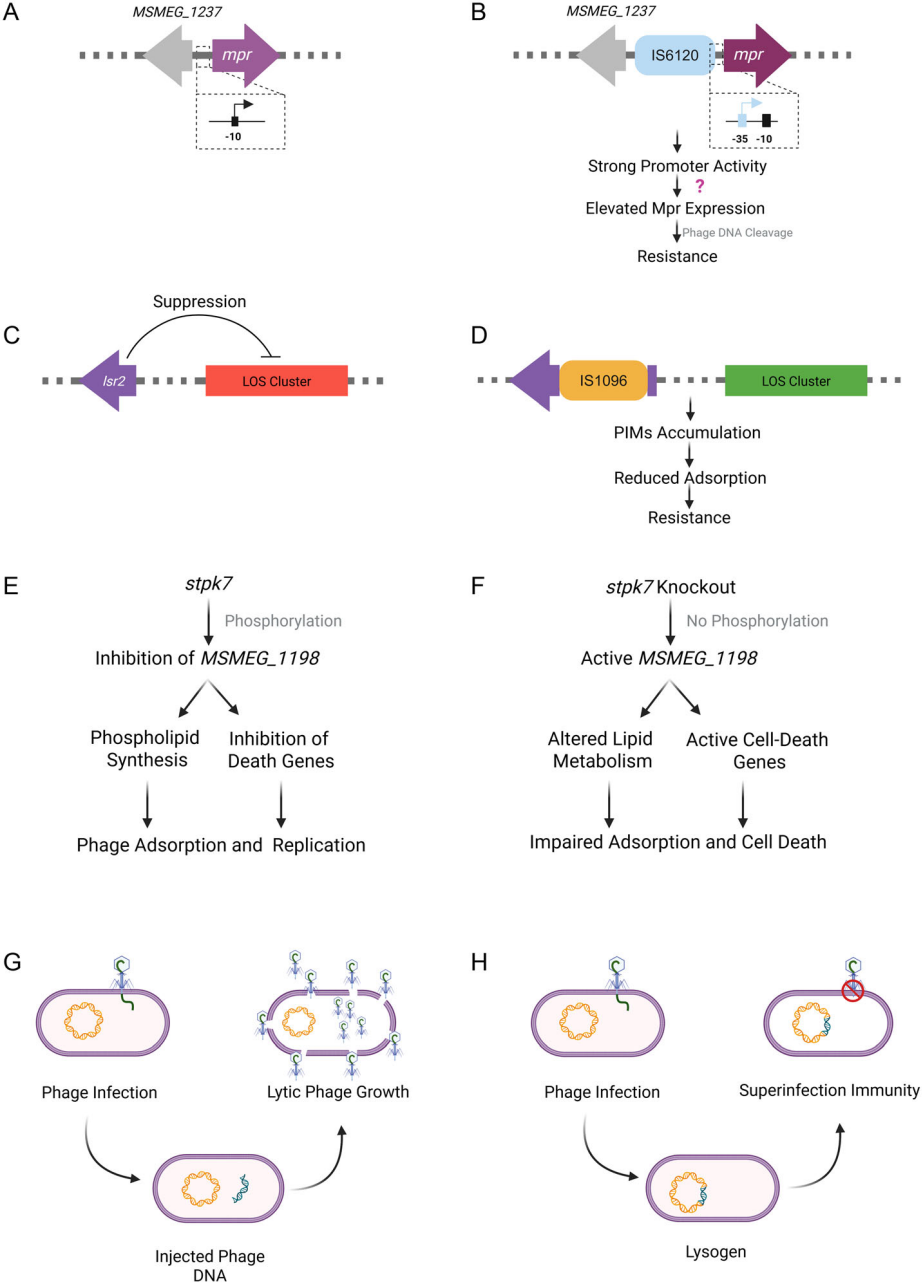
Representative mycobacterial antiphage defense systems. Panels on the left (A,C,E,G) represent susceptible (or wild‐type) backgrounds, whereas those on the right (B,D,F,H) represent the phage‐resistant backgrounds. The multicopy phage resistance protein (Mpr) in *M. smegmatis*, when overexpressed, mediates resistance to the mycobacteriophage D29 (A,B). This overexpression‐driven resistance mechanism is not due to any mutation within *mpr* itself but rather depends on overexpression of a wild‐type copy of the gene (Barsom and Hatfull 1996). Spontaneous D29‐resistant mutants of *M. smegmatis* with elevated Mpr expression have been reported to sustain the transposition and integration of the insertion sequence element IS6120 upstream of *mpr*, which introduces a transcription factor‐binding site and a canonical −35 promoter element at the integration site, hence reconstituting a more complete, powerful promoter directly upstream of *mpr* (Yusuf et al. 2026). This is likely responsible for elevated expression of *mpr*, which encodes a membrane‐bound DNA exonuclease that cleaves D29 DNA during injection (Seniya and Jain 2022). Similarly, transposition and integration of IS1096 into the negative transcriptional regulator *lsr2* activates the *M. smegmatis* LOS cluster, resulting in accumulation of PIMs on the membrane and defective phage adsorption (C,D) (Li et al. 2024). StpK7 is a serine/threonine kinase that, when intact, phosphorylates and inhibits the positive regulatory effect of MSMEG_1198 on the BREX‐like gene island *MSMEG_1191‐1200*, hence enabling the mycobacteriophage TM4 to establish successful infection in *M. smegmatis* (E). Deletion of *stpK7* allows for MSMEG_1198 activity, hence activating defensive genes (F) (Li et al. 2023). In the absence of prophage‐mediated antiphage defense, phages can successfully infect and kill their bacterial hosts (G). However, prophage elements–for example, from cluster N mycobacteriophages–confer their lysogens with homotypic/heterotypic phage resistance, hence enabling the host to resist subsequent infection by another phage that may/may not be related to the resident prophage (H) (Mageeney et al. 2020; Dedrick et al. 2017). Figure was created in BioRender by Malik, A. (2026): https://BioRender.com/4nmq09i.

Despite the vast number of bacterial phage resistance mechanisms, only a few have been described in mycobacteria (Figure 2). Prophages drive diverse mechanisms of defense against bacteriophages (Bondy‐Denomy et al. 2016), including in mycobacteria (Mageeney et al. 2020; Dedrick et al. 2017; Gentile et al. 2019; Mohammed et al. 2023). Examples of prophage elements that drive homotypic/heterotypic defenses against lytic and temperate mycobacteriophages are the Panchino *gp28* restriction system, the Charlie *gp32* heterotypic exclusion system, Butters *gp30* and *gp31* membrane complex, and a predicted (p)ppGpp synthetase (Phrann *gp29*) (Mageeney et al. 2020; Dedrick et al. 2017). Lysogenization may therefore jeopardize the effectiveness of phage therapy by conferring the bacterial pathogen with homotypic/heterotypic phage resistance, which explains why lysogenic/temperate phages may not be good therapeutic candidates without prior engineering.

Changes to lipid profile are also associated with phage resistance in mycobacteria—for example, glycopeptidolipids, lipooligosaccharides (LOS), and phosphatidylinositol mannosides (PIMs) in *M. smegmatis*—raising the question of whether some of these lipids are actual host surface receptors (Besra et al. 1994; Dhariwal et al. 1986; Furuchi and Tokunaga 1972; Imaeda and Blas 1969; Wetzel et al. 2023; Li et al. 2024; Li et al. 2023; Chen et al. 2009). O‐acylation and O‐methylation of LOS have been reported in mycobacteriophage D29‐resistant strains of *M. smegmatis*, raising the question of whether these modifications could explain the chemical basis of phage lysogenization or resistance in mycobacteria (Besra et al. 1994). The *M. smegmatis* LOS biosynthesis cluster is negatively regulated by Lsr2 (Kołodziej et al. 2021; Etienne et al. 2009). Integration of IS1096 into *lsr2* activates the LOS biosynthesis cluster by relieving it of the *lsr2*‐driven repression, resulting in elevated synthesis and accumulation of PIMs on the cell envelope and defective phage adsorption (Li et al. 2024). Strain morphotype also strongly correlates with phage susceptibility or resistance in *Mycobacterium abscessus* clinical isolates, with higher susceptibility and a rare chance of resistance seen in rough strains, further emphasizing the relevance of lipid homeostasis in phage‐mycobacteria interactions (Dedrick et al. 2021).

Upon overexpression, the gene *mpr* confers *M. smegmatis* with resistance to the closely‐related mycobacteriophages L5 and D29 (Barsom and Hatfull 1996). Mpr is a membrane‐bound exonuclease that mediates antiphage defense by blocking entry of D29 DNA through the membrane of *M. smegmatis* (Seniya and Jain 2022), hence blocking downstream stages in the phage infection cycle. Multiple copies of *mpr* are necessary for phage resistance, and under normal growth conditions, only low or even undetectable amount of the protein is expressed by *M. smegmatis*. In addition, phage infection itself does not trigger Mpr overexpression, which suggests that this gene plays a biological function that remains to be uncovered (Seniya and Jain 2022). However, we uncovered that insertion sequence rearrangement likely constitutes the genetic basis for Mpr overexpression in spontaneous D29‐resistant mutants of *M. smegmatis* (Yusuf et al. 2026). We observed that the insertion element IS6120 integrates 60 bp upstream of *mpr* in these mutants, which elevated Mpr expression by multiple folds. Further evaluation revealed that IS6120 introduced the −35 promoter element “TTGACA” at the integration site, which is absent in the wild‐type strain. This strengthened the promoter and likely drives higher expression of Mpr to defend against phage invasion. Since a high level of Mpr expression is toxic to the bacteria, this mechanism of regulation by promoter reconstitution implies that the bacteria tightly regulate Mpr expression.

Bacteriophage exclusion (BREX) system is a bacterial antiphage defense system that allows for adsorption but inhibits phage replication, and distinct classes of these as well as other BREX‐like systems are widespread in microbial genomes. This system works by introducing m^6^A methylation on the 5^th^ nucleotide in the nonpalindromic motif 5′‐TAGGAG‐3′ across the genome of the host bacterium to distinguish self from nonself, and unlike restriction‐modification systems, BREX doesn’t appear to cleave/degrade phage DNA (Goldfarb et al. 2015; Chaudhary 2018). *M. smegmatis* genome harbors a 10‐gene cluster spanning *MSMEG_1191* through *MSMEG_1200*, a so‐called BREX‐like island. This gene island is associated with lipid homeostasis and also determines susceptibility of *M. smegmatis* to the mycobacteriophage TM4. Deletion of *MSMEG_1200* (*stpK7*) leads to TM4 resistance, and this resistance phenotype requires the remaining 9 genes in the cluster. Following phage infection, intact StpK7 in the bacteria negatively affects the expression of other genes in the cluster by phosphorylating and inhibiting the positive regulatory activity of MSMEG_1198, thereby suppressing cell death genes and ensuring bacterial survival, which favors phage adsorption and proliferation (Li et al. 2023).

## 4 Fitness Trade‐Offs Due to Phage Resistance

Bacterial resistance to phages could be complete or partial and usually comes at high or low fitness costs (Vasu and Nagaraja 2013; Vale et al. 2015; Azam and Tanji 2019; Sørensen et al. 2021). Because the evolution of bacterial resistance to phages could involve mutations in genes encoding important molecules, phage‐resistant bacteria could suffer a significant loss of fitness. For example, bacteria may suffer a significant reduction in virulence if the mutated gene encodes important surface lipids such as lipopolysaccharides or exhibit reduced growth if the mutations occur in genes that encode proteins required for essential functions (Burmeister and Turner 2020; McGee et al. 2023).

Trade‐offs in the form of high fitness costs result from bacterial efforts to defend against phages, including trading off virulence determinants or phenotypes such as biofilm formation and AMR (McGee et al. 2023; Li et al. 2022; Lerouge and Vanderleyden 2002). Combined phage/antibiotic therapy could therefore take advantage of these trade‐offs to improve treatment of drug‐resistant bacterial infections (Bao et al. 2020). In fact, previous studies investigating the utility of this approach have yielded promising results (Aslam et al. 2019; Tagliaferri et al. 2019). It is thought that in case of antibiotic resistance trade‐off for phage resistance, sequential (not simultaneous) phage and antibiotic therapy could improve treatment and avoid emergence of both phage and antibiotic resistance in the bacteria (McGee et al. 2023).

Specific antiphage defenses could be countered by corresponding mutations in the infecting phage, enabling the phage to evade host defense and kill the host in a process called defense escape (Bohannan and Lenski 2000; Weissman et al. 2018). Therefore, defense escape could be leveraged in phage therapy, provided the phage resistance phenotype could be overcome. For example, coevolutionary phage training (discussed in the next section) is one way of leveraging defense escape for therapeutic advantage. Infection with a trained phage delays the evolution of bacterial resistance and triggers the accumulation of multiple mutations in the host as a defensive response. Each of these mutations may come at a great fitness cost compared to single mutations that arise as a result of exposure to naïve (untrained) phage. It is therefore thought that trained phage and host immune system could leverage the reduced fitness of the bacteria to resolve bacterial infections (Borin et al. 2021).

## 5 Coevolutionary Phage Training

Just like AMR‐conferring mutations, mutations associated with phage resistance are often common (LeClerc et al. 1996; Oliver et al. 2000; Luria and Delbrück 1943), including those involved in cross‐resistance to multiple phages (Wright et al. 2018). However, the biological nature of phages offers them a great level of malleability. This is to say that, unlike antibiotics, phages can respond to changes in their hosts in order to adapt and retain their ability to infect the host, provided the host defense mechanism can be overcome. This coevolution process is often termed “arms race” between phages and their bacterial hosts, in which the host develops antiphage defenses, whereas the phage reciprocally changes to find ways of counter‐attack/evading host defenses. Coevolution could therefore be harnessed to improve the effectiveness of phage therapy via “coevolutionary phage training,” which has been proposed as a potential approach to preemptively counter the evolution of bacterial resistance to phages (Rohde et al. 2018; Laanto et al. 2020; Woolhouse et al. 2002). It involves infecting the target host with the phage such that both coevolve together, during which changes occur in the bacterial host in response to phage stress, whereas the phage adapts by developing counter‐attack measures. This evolved phage is termed a “trained phage,” which could be used to target an uncoevolved/naïve bacteria in a patient. Because the trained phage has evolved possible counter‐attack measures against the target bacteria, evolution of resistance by the target bacteria is delayed or rendered futile (Borin et al. 2021; Laanto et al. 2020; Monferrer and Domingo‐Calap 2019; Peters et al. 2020).

However, there are diverging theories regarding the dynamics of phage‐host coevolution (Torres‐Barceló 2018; Woolhouse et al. 2002; Hall et al. 2011). While some argue that a phage adapted to its evolving host loses the ability to infect past hosts, others argue that it maintains its ability to do so (Woolhouse et al. 2002). It is also thought that phage training would only accelerate the evolution of phage resistance by mounting stronger selective pressure on the target host (Rohde et al. 2018). Together, these cast doubt on the potential utility of phage training to improve the effectiveness of phage therapy. However, the coevolution study of *E. coli* and a lytic strain of *λ* phage reveals that phage training could hold the potential to improve phage therapy outcome (Borin et al. 2021). It reveals that while a single mutation could confer resistance to an untrained phage, multiple mutations acquired over a prolonged period are required to confer resistance to a trained phage, indicating extended suppression of the bacteria by a trained phage. In fact, single mutations were ∼100‐fold more common in naïve (uncoevolved) cells treated with an untrained phage and could only confer partial resistance against a trained phage. In addition, while the untrained phage uses only LamB as a receptor, the trained phage uses both LamB and OmpF as receptors, suggesting that coevolutionary training enabled the phage to recognize an additional receptor to improve infection. In addition, phage resistance carryover has been demonstrated in *E. coli*, in which phage‐resistant strains were shown to be resistant to other related phages with overlapping receptor requirements (McGee et al. 2023). This could therefore present an impediment to the implementation of phage therapy. To address this, using a cocktail of phages with varying adsorption mechanisms could maximize the effectiveness of phage therapy against a particular bacterial host. This represents an interesting development as phages that use backup receptors would yield a greater outcome than their single receptor‐recognizing counterparts.

## 6 Mycobacteriophage Therapy

The profound threat posed by drug‐resistant bacterial infections, including those caused by mycobacteria, raises the need for efficient preventive measures, a rich and effective drug pipeline, as well as the development of alternatives to conventional antibiotic therapy. However, these efforts are faced with certain hurdles that limit the progress in the prevention and control of infectious diseases. For example, development of drug resistance by pathogenic bacteria outpaces the rate at which new antibiotics/vaccines are introduced into clinical use, and it has been over a century since the discovery of the only vaccine currently available for use against TB–the *Bacille Calmette–Guérin* (BCG). This warrants the outlook for potential alternatives to widen treatment options for bacterial infections, especially those involving drug‐resistant strains.

Mycobacteriophage therapy is the use of mycobacteriophages to treat mycobacterial infections. Currently, clinical use of bacteriophages is accessible under the United States Food and Drug Administration (USFDA) policy of expanded access (or compassionate use). Under serious or immediately life‐threatening conditions, and in the absence of any comparable or satisfactory therapeutic alternative, expanded access allows for clinical use of an investigational medical product outside of clinical trials (Expanded Access | FDA 2025). Generally, expanded access is deemed appropriate when the case meets all of the following: serious or immediately life‐threatening condition; absence of comparable or satisfactory option; likely benefit of compassionate use justifies the possible risk; it is not possible to enroll the patient in a clinical trial; and compassionate use will not interfere with trials that could back development or approval for an investigational product. Under this framework, therapeutic usage of mycobacteriophages against mycobacterial infections has so far yielded varying clinical outcomes (Dedrick et al. 2019; Cristinziano et al. 2024; Dedrick et al. 2023; Dedrick et al. 2021; Nick et al. 2022; Little et al. 2022; Dedrick et al. 2022; Jernigan and Hentish 2025). Nevertheless, given that phage therapy is applied only based on compassionate use, the results represent a significant progress in the treatment of drug‐resistant mycobacterial infections.

Here is a timeline of reports of clinical use of mycobacteriophages to treat mycobacterial infections: 2019—in a 15‐year old (yo) patient with disseminated *M. abscessus* infection following a bilateral lung transplant; 2021 & 2022—in an 81‐yo patient with bronchiectasis caused by *M. abscessus*; 2022—in a 26‐ and a 56‐yo patients with lung (*M. abscessus*) and cutaneous (*M. chelonae*) infections respectively; 2023—in 20 pediatric and adult patients with localized or disseminated infections caused by *M. abscessus*, *M. chelonae*, *M. avium* complex, or BCG; 2024—in an elderly patient in their 60s with sternal osteomyelitis and soft tissue infection caused by *M. abscessus*; 2025—in a 45‐yo patient with multimicrobial activation syndrome (MMAS) and noncystic fibrosis (CF) bronchiectasis caused by a polymicrobial infection; and 2026—in a 25‐ and a 47‐yo patients with CF and bronchiectasis respectively, with both patients having *M. abscessus* infection localized to the thoracic region (Table 1) (Dedrick et al. 2019; Cristinziano et al. 2024; Dedrick et al. 2023; Dedrick et al. 2021; Nick et al. 2022; Little et al. 2022; Dedrick et al. 2022; Jernigan and Hentish 2025; Ferreiro‐Posse et al. 2026). All patients, except the 2025 case, received phage therapy as an adjunct to antibiotic therapy. In all of the 2019–2024 and the 2026 cases, therapy involved the use of a single or a combination of wild‐type, host range mutant, or engineered mycobacteriophages. Induced native phage therapy (INPT) was used in the 2025 case, where more than 30 microbes (including *M. avium* complex, *M. abscessus*, *M. tuberculosis*, *M. kansasii*, and *M. intracellularis*) were targeted in a single induced native phage cocktail formulation. A total of 28 patients (at least 27 certainly known to be infected by mycobacteria) received phage therapy for mycobacterial infections, where *M. abscessus* accounts for 82.1% of all cases in contrast to *M. chelonae* (7.1%), *M. avium* complex (3.6%), BCG (3.6%), and the polymicrobial infection (3.6%). When an infection involves both morphotypes of *M. abscessus*, the smooth morphotype has been shown to persist, even in cases where both have identical drug susceptibility profiles. This stems from varying susceptibilities of the two morphotypes to phage infection (Cristinziano et al. 2024; Dedrick et al. 2021; Dedrick et al. 2023).

**TABLE 1:** Timeline of global reports of mycobacteriophage therapy.

Year	Age^a^	Organism^b^	Condition^c^	Organ		Phage(s)	Route^d^	Duration		Outcome	Resistance^e^	References
2019	Pd	*M. abscessus*	CF, bilateral lung transplant	Disseminated infection		Muddy, BPs^f^, ZoeJ^f^	IV	8 months		Favorable	No	Dedrick et al. (2019)
2021	Ad^g^	*M. abscessus*	Bronchiectasis	Lung		Muddy, BPs^f^, ZoeJ^f^	IV	6 months		Limited efficacy	Yes	Dedrick et al. (2021)
2022	Ad^g^	*M. abscessus*	Bronchiectasis	Lung		Muddy, BPs^f^, ZoeJ^f^	Neb	9 months		Transient improvement	No	Dedrick et al. (2022)
	Ad	*M. abscessus*	CF, bronchiectasis	Lung		BPs^f^, D29^f^	IV	1.6 years		Favorable resolution	No	Nick et al. (2022)
	Ad	*M. chelonae*	Seronegative arthritis	Cutaneous		Muddy	IV	>6 months		Stable improvement	No	Little et al. (2022)
2023	Pd	*M. abscessus*	CF, bilateral lung transplant	Disseminated infection		Muddy, BPs^f^, ZoeJ^f^	IV	3.5 years^h^		Improved	No	Dedrick et al. (2023)
	Ad	*M. abscessus*	Scleroderma, lung transplant	Lung, sternal bone infection		Muddy	IV, chest wash	1 month^h^		Improved	No	
	Pd	*M. abscessus*	CF, lung transplant	Lung		BPs^f^	IV	1 month^h^		Improved	No	
	Pd	*M. abscessus* (S and R)	CF	Lung		BPs^f^	IV	7 months^i^		Partially improved	No	
	Pd	*M. abscessus*	CF	Lung		Muddy, BPs^f^	IV, bronchoscopic administration	1 year		Favorable resolution	No	
	Ad	*M. abscessus*	CF, lung transplant	Lung, disseminated infection		BPs^f^, Itos	IV	1 year		Favorable resolution	No	
	Ad	*M. abscessus*	CF	Lung		Muddy	IV, Neb	11 months		Improved	No	
	Ad	*M. abscessus*	CF	Lung		BPs^f^, D29^f^	IV	∼1 year		Favorable	No	
	Ad	*M. chelonae*	Seronegative arthritis	Disseminated cutaneous infection		Muddy	IV	9 months		Favorable resolution	No	
	Pd	*M. avium* complex	CF	Lung		Muddy	IV, Neb	3 months		Improved	No	
	Pd	BCG	MSMD	Disseminated infection		Muddy, D29, Fionnbharth^f^, Fred313cpm^f^	IV	1.7 years^h^		Improved	—	
	Ad	*M. abscessus*	CF	Lung		BPs^f^, Itos	IV	10 days^h^		Deceased	—	
	Ad	*M. abscessus*	CF	Lung		Muddy	IV, Neb	1.3 years		Inconclusive (improving)	No	
	Pd	*M. abscessus* (S and R)	CF	Lung		Muddy	IV	1.7 years		Inconclusive (improving)	No	
	Ad	*M. abscessus*	Chronic lung bronchiectasis	Lung		Muddy, BPs^f^, ZoeJ^f^	IV, Neb	1.6 years^h^		Improving	Partial	
	Ad	*M. abscessus*	CF	Lung		Muddy	IV, Neb	4 months		Little improvement	No	
	Ad	*M. abscessus*	CF	Lung		Muddy	IV, Neb	1.1 year		No substantial improvement	No	
	Ad	*M. abscessus*	CF	Lung		BPs^f^	IV	6 months		No substantial improvement	No	
	Ad	*M. abscessus*	CF	Lung		BPs^f^, D29^f^	IV	11 months		No clinical improvement	—	
	Ad	*M. abscessus*	CF	Lung		BPs^f^, Itos	IV, Neb	∼6 months		No substantial improvement	No	
2024	Ad	*M. abscessus* (S and R)	Sternal osteomyelitis and soft tissue infection	Lung		Muddy, BPs^f^	IV	∼1 year		Improved	No	Cristinziano et al. (2024)
2025	Ad	Polymicrobial	MMAS and severe non‐CF bronchiectasis	Lung		Induced native phage cocktail formulation (Inducen‐Res)	Oral	6 months		Favorable resolution	No	Jernigan and Hentish (2025)
2026	Ad	*M. abscessus*	CF	Lung chest wall		BPs^f^, Itos	IV	12 months		Improved	Yes	Ferreiro‐Posse et al. (2026)
	Ad	*M. abscessus*	Bronchiectasis	Lung		BPs^f^, D29^f^	IV	12 months^h^		Improved	No	
**Favorable/improved outcomes**					**Complicated/inconclusive/unsatisfactory outcomes**				**Cases with no obvious improvement**			

a Patient age is indicated as Pediatric (Pd) or Adult (Ad).

b This is the mycobacterial species involved in the infection in the indicated patient. For *M. abscessus*, it is indicated whether the patient has infection involving both Smooth (S) and Rough (R) morphotypes.

c CF, cystic fibrosis; MMAS, multimicrobial activation syndrome; MSMD, Mendelian susceptibility to mycobacterial disease.

d Routes of administration are intravenous (IV), nebulization (Neb), or a combination of both during the treatment course.

e This indicates whether phage resistance was detected during the treatment course.

f This phage is a host range mutant or has been engineered for the purpose of treatment.

g This is the same patient who experienced limited therapeutic efficacy due to robust IgM‐ and IgG‐mediated phage neutralization. The route of administration was changed to nebulization according to the 2022 report, but the treatment ultimately failed with only transient improvement. Phage resistance was detected, but no *M. abscessus* isolate from the patient was resistant to all three phages in the cocktail.

h Patient died due to nonphage related complications.

i Culture conversion to negative for the rough morphotype, but the smooth morphotype persisted.

### 6.1 Lessons Derived from Case Reports of Mycobacteriophage Therapy

Drug‐resistant mycobacterial infections pose a unique public health threat, especially those involving nontuberculous mycobacteria (NTM). This is owed to the innately high drug resistance profiles of these pathogens. Though we did not find a case report of mycobacteriophage therapy for TB, there are ongoing efforts to study the usability of mycobacteriophages against *Mycobacterium tuberculosis* under disease‐relevant conditions (Janssen et al. 2026). At least 26 of the 28 patients reported to have received mycobacteriophage therapy had infections involving NTM, with *M. abscessus* alone accounting for 21 of the cases (Table 1). It could therefore be suspected that this is so partly due to narrower therapeutic options for complex NTM infections compared to infection involving *M. tuberculosis*.

We learn from these case reports that even in complex cases of mycobacterial infections, including failed antibiotic therapy, phage therapy could offer a viable alternative. Though treatment was still ongoing in some patients when the reports were published, the course of mycobacteriophage therapy ranged from a few months to more than 3 years (Table 1). The duration and sustainability of phage therapy, particularly in high AMR settings, may be influenced by several known and unknown factors, which may include the nature of the infection itself (localized or disseminated), the availability of a suitable therapeutic phage, antibody‐mediated phage neutralization, evolution of phage resistance, sensitivity/resensitization of the pathogen to antibiotic therapy, phage‐antibiotic synergy, and patient's tolerance to therapy.

Many of the patients had shown significant clinical improvements or even resolved infections. Three patients had lung infections involving both smooth and rough morphotypes of *M. abscessus*, and treatment of these patients presented more challenge than infections involving only the rough morphotype (Cristinziano et al. 2024; Dedrick et al. 2023). This is because the rough morphotype tends to be cleared more easily by phage therapy, whereas the smooth morphotype tends to persist even when both have identical drug susceptibility profiles (during combined phage and drug therapy), which stems from varying susceptibility of smooth and rough morphotypes of *M. abscessus* to phage infection (Dedrick et al. 2021).

**FIGURE 3:**
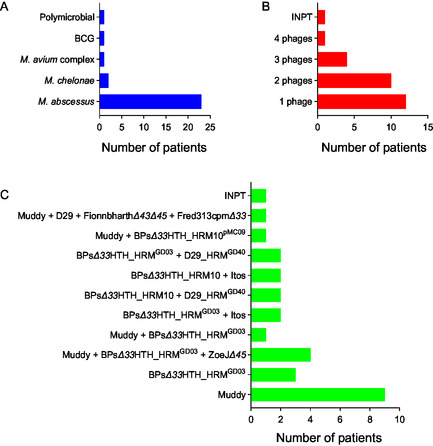
Isolates involved in mycobacterial infections and cocktails used in mycobacteriophage therapy. (A) At least 26 of the 28 patients that received mycobacteriophage therapy had NTM infections, and a vast majority of them involved *M. abscessus*: 1 patient each had BCG, *M. avium*, or polymicrobial infection; 2 had *M. chelonae* infections; and 21 had *M. abscessus* infections. (B) Twelve of the patients were treated with only one phage. The number of phages in each cocktail ranged from two to four, with most treated with two‐ or three‐phage cocktails. Only the BCG‐infected patient was treated with a four‐phage cocktail, and one patient received INPT. Thirty microbes (including *M. avium* complex, *M. abscessus*, *M. tuberculosis*, *M. kansasii*, and *M. intracellularis*) were targeted in the induced native phage cocktail formulation. (C) The different phages/cocktails and the number of patients that were treated with them. Muddy appears to be a good therapeutic candidate. Most phages, however, are either host range mutants or were subjected to genetic/epigenetic modifications to boost their therapeutic potentials (Dedrick et al. 2019; Cristinziano et al. 2024; Dedrick et al. 2023; Dedrick et al. 2021; Nick et al. 2022; Little et al. 2022; Dedrick et al. 2022; Jernigan and Hentish 2025).

Identifying a suitable phage/phage cocktail could be challenging in certain cases, requiring isolation of host range mutants or engineering the phages via introduction of genetic/epigenetic modifications (Dedrick et al. 2019; Cristinziano et al. 2024). This is even more so when the infection involves the smooth morphotype of *M. abscessus*, which has limited susceptibility to phage infection in contrast to its rough counterpart. Collectively, 10 host range mutants, engineered and wild‐type mycobacteriophages (Muddy, BPsΔ*33*HTH_HRM^GD03^, ZoeJΔ*45*, Itos, BPsΔ*33*HTH_HRM10, D29_HRM^GD40^, BPsΔ*33*HTH_HRM10^pMC09^, D29, FionnbharthΔ*43*Δ*45*, Fred313cpmΔ*33*) with proven clinical utility have been identified against *M. abscessus*, *M. chelonae*, *M. avium* complex, and BCG infections (Table 1 and Figure 3). In addition, bacterial strains isolated from the patients rarely developed phage resistance. Though this represents a good leap, it still warrants more efforts in building a robust pipeline of therapeutically useful phages through the identification of novel phages and expanding the host range of existing ones.

So far, patients who received phage therapy were treated with at least a single phage and at most a four‐phage cocktail (Figure 3). In fact, 12 of the 28 patients were treated with either Muddy or a host range mutant of the lytic derivative of the mycobacteriophage BPs (BPsΔ*33*HTH_HRM^GD03^), and no phage resistance was observed. Another phage cocktail that was found suitable for multiple patients is the combination of Muddy, BPsΔ*33*HTH_HRM^GD03^, and an engineered derivative of ZoeJ (ZoeJΔ*45*). Use of these and other cocktails in the patients yielded impressive results, given the conditions of these patients before treatment. This certainly constitutes a step forward in the fight against drug‐resistant mycobacterial infections.

Treatment was administered either intravenously (IV) or by nebulization (Neb) or both in some cases. An exception is INPT, where the induced native phage cocktail formulation was administered via the oral route. The IV method is most commonly used, though there was a transition to Neb in the 81‐yo patient when robust antibody responses were detected.

A number of patients encountered adverse events, which were not attributed to phage therapy. For example, despite improved responses to phage therapy, some patients succumbed to their infections due to nonphage‐related complications such as underlying coinfections, multiple transplant complications, adenovirus systemic infection, and multiple organ failure (Dedrick et al. 2023). Phage therapy failed in the 81‐yo patient due to robust IgM‐ and IgG‐mediated antibody responses (Dedrick et al. 2021; Dedrick et al. 2022). However, in the 26‐yo and the 56‐yo patients, stable improvements, including favorable resolution of infection, were recorded despite antibody responses (Nick et al. 2022; Little et al. 2022).

Phage therapy is essentially personalized therapy and often requires screening, engineering, or adapting the candidate phages to suit the particular strain isolated from the patient. This may delay access to phage therapy, especially under serious, life‐threatening situations. In such cases, INPT, a form of generalized phage therapy, may come in handy. Inducen‐Res, an induced native phage cocktail formulation, resolved a polymicrobial infection in a 45‐yo patient with MMAS and severe non‐CF bronchiectasis. More than 30 microbes (including *M. avium* complex, *M. abscessus*, *M. tuberculosis*, *M. kansasii*, and *M. intracellularis*) were targeted in the Inducen‐Res formulation, and it was reported to resolve the infection after 6 months of treatment (Jernigan and Hentish 2025).

### 6.2 Impact of Host Immunity on Mycobacteriophage Therapy

Studies in macrophage‐ablated larvae of zebra fish (Johansen et al. 2021) and neutrophil‐depleted mice (Roach et al. 2017) suggest that functional innate immunity is crucial in ensuring effective phage therapy. Likewise, mycobacteriophage therapy is associated with complex immune reprogramming through differential regulation of both innate and adaptive immunity in humans, including elevated expression of proinflammatory and immune‐activating factors (e.g., CXCL10, IL‐8, TIFA, TRAT1, IDO1) as well as those involved in stress adaptation and tissue remodeling (e.g., HSP90AA1, CCNC, SELENOS, and NAE1). These responses are believed to promote efficient pathogen containment and clearance, recovery of tissue integrity, and maintenance of homeostasis in the face of chronic infection and inflammation (Ferreiro‐Posse et al. 2026).

The body produces neutralizing antibodies in response to phage administration but only in a few cases did this seem to render the mycobacteriophages ineffective. Though antibody response is suspected to limit therapeutic efficacy in a few cases, treatment was effective in most cases (Dedrick et al. 2023; Little et al. 2022; Dedrick et al. 2022). Common antibody responses to phage therapy include IgG, IgM, and IgA, but IgG and IgM responses were mostly stronger compared to IgA responses (Dedrick et al. 2023; Dedrick et al. 2021; Nick et al. 2022; Little et al. 2022; Dedrick et al. 2022; Gembara and Dąbrowska 2021; Wannigama et al. 2025). Phage neutralization is weaker in the sputum than in blood sera, suggesting that the impact of host immunity on phage viability may vary with the route of phage administration (Dedrick et al. 2021; Dedrick et al. 2022; Wannigama et al. 2025; Łusiak‐Szelachowska et al. 2014; Berkson et al. 2024). In fact, it is suggested that noncontinuous or serial administration of phages may be desirable in cases of extended intravenous administration to avoid all phages being neutralized at once by host antibodies (Dedrick et al. 2021).

Poor bacterial clearance in patients with compromised immunity is in part influenced by poor efficiency of the immune system, hence enabling the bacteria to persist, including under antibiotic pressure (Alkhater 2009). However, immunosuppressed individuals may have a greater level of tolerance to prolonged phage therapy without mounting strong phage‐neutralizing antibody responses. Neutralizing responses appear to limit phage therapy efficacy in an immunocompetent patient (Dedrick et al. 2021) but not in an immunosuppressed patient (Dedrick et al. 2019), raising the question of whether immunosuppression may avert the production of phage‐neutralizing antibodies, hence preserving phage efficacy. Under immunosuppressed conditions, weak antibody responses to phage proteins are detected with no evidence of phage neutralization. There were also weak cytokine responses after treatment initiation, including interferon‐γ (IFNγ), interleukin‐6 (IL‐6), IL‐10, and tumor necrosis factor‐α (TNFα) (Dedrick et al. 2019). Under immunocompetent situations, however, antibody responses may limit the efficacy of phage therapy due to robust IgM‐ and IgG‐mediated phage neutralization (Dedrick et al. 2021). Therefore, host response to phage therapy may vary between immunocompetent andimmunosuppressed individuals, and different therapeutic approaches may be required under these situations (Dedrick et al. 2019; Cristinziano et al. 2024; Dedrick et al. 2023; Dedrick et al. 2021; Ferreiro‐Posse et al. 2026). Moreover, antibody‐mediated phage neutralization can also occur in immunosuppressed individuals, and some immunocompetent individuals have demonstrated lack of antibody‐mediated phage neutralization (Dedrick et al. 2023), hence suggesting the likely influence of other unrecognized factors on phage therapy (Nick et al. 2022).

Antibody‐mediated phage neutralization results from independent immune reactions to each specific phage in the cocktail rather than cross‐reactivity (Dedrick et al. 2021), and the strength of each response may vary with the phage itself, raising the question of whether some phages may be more immunogenic than others (Dedrick et al. 2021; Nick et al. 2022). For example, in one patient, neutralization and inactivation of phage BPsΔ33HTH_HRM10 was observed, though there was a sputum culture conversion to negative before the development of significant neutralizing antibody responses. However, only mild neutralization of phage D29_HRM^GD40^ was observed, and the phage remained active throughout the treatment period (Nick et al. 2022).

Individuals may have pre‐existing, cross‐reactive antibody responses against the therapeutic phages before treatment initiation, which may suggest prior exposure to the same or related phages. These responses, however, may not be phage neutralizing and therefore should not be considered as contraindicative (Dedrick et al. 2023; Dedrick et al. 2021; Nick et al. 2022; Little et al. 2022).

Therefore, while antibody responses may have a temporal correlation with clinical response to phage therapy, they often have no correlation with clinical outcome, as evident in favorable clinical outcomes in several patients despite antibody responses (Dedrick et al. 2023). Other potential impacts of host immunity on phage therapy have been extensively reviewed elsewhere (Sithu Shein et al. 2024).

### 6.3 Benefits and Limitations of Phage Therapy

While prolonged antibiotic therapy may be toxic and/or favors the evolution of multidrug‐resistant bacteria, phage therapy is relatively safe and tolerable, with a remarkable success rate, minimal to no toxicity, and absence of phage‐related adverse reactions (GBD 2021; Dedrick et al. 2023; Sithu Shein et al. 2024; Wienhold et al. 2021). It is also highly specific, with no effect on beneficial microbiota or mammalian cells. It resensitizes bacteria to antibiotics, hence enhancing the clearance of drug‐resistant bacteria (Ferreiro‐Posse et al. 2026; Johansen et al. 2021; Chow et al. 2020). Phages can dissolve biofilms to reach infections, hence increasing drug penetration and improving the effectiveness of antibiotic therapy (Shahed‐Al‐Mahmud et al. 2021; Henriksen et al. 2019). Induced native phage therapy can be tailored to target not just the microbial pathogens but also their associated exotoxins, endotoxins, and lipopolysaccharides to enhance bacterial killing (Jernigan and Hentish 2025).

Phage therapy triggers immune reprogramming by stimulating cytokine production and macrophage activation. Phage‐mediated bacterial lysis exposes pathogen‐associated molecular patterns, which enhance innate immune recognition and TLR signaling, hence facilitating pathogen containment and clearance (Ferreiro‐Posse et al. 2026; Cui et al. 2024; Venturini et al. 2022). When antibiotics and phages are used together, their combined effects provide greater effectiveness than phage‐ or drug‐only therapy by lowering bacterial burden, reducing severity of infection, and improving survival (Johansen et al. 2021; Chow et al. 2020; Jeon and Yong 2019; Liu et al. 2022; Gu Liu et al. 2020; Gurney et al. 2020; Zhang et al. 2021; Debarbieux et al. 2010; Duplessis et al. 2021; Lin et al. 2021). Though clinical use of amikacin and D29_HRM^GD40^ within the same treatment course yielded favorable outcomes (Nick et al. 2022; Ferreiro‐Posse et al. 2026), future studies may consider investigating the clinical implications of drug antagonism to phage replication (Jiang et al. 2020).

In cases where bacterial resistance to phages arises, resistance‐associated fitness trade‐offs may increase bacterial susceptibility to drugs as well as immune clearance (Menon et al. 2022). Benefits uncovered during preclinical studies have been extensively reviewed elsewhere (Sithu Shein et al. 2024), which include reduced lung inflammation (Zhang et al. 2021; Yang et al. 2015), rapid resolution of immune response (Lin et al. 2021), improved histopathological outcome (Oduor et al. 2016; Prazak et al. 2019), and reduced levels of inflammatory cytokines (Chang et al. 2022).

Major limitations include the failure to isolate phages with broad‐spectrum activity against clinical strains isolated from all patients, which makes it necessary to screen and identify a suitable phage candidate for each case (Dedrick et al. 2023). Phage‐specific neutralizing immune responses limit phage efficacy, and use of related phages in a cocktail may elicit cross‐reactive antibody responses. However, understanding of the potential reasons for treatment failure would be invaluable in devising ways of overcoming these hurdles. For example, phage engineering, phage surface modification, and coadministration with immunosuppressive agents have been suggested as potential strategies to circumvent the effects of phage‐neutralizing antibodies (Singla et al. 2016; Kaur et al. 2021; Wang et al. 2024). Therefore, significant clinical research efforts ought to go into uncovering viable ways of circumventing the effects of phage‐neutralizing antibodies while maintaining safety and phage efficacy.

Individuals may have pre‐existing, cross‐reactive antibody responses against the therapeutic phages before treatment initiation, which may suggest prior exposure to the same or related phages. These responses may increase either mildly or significantly over the treatment course and may have a significant impact on treatment efficacy. If these responses persist, they may also affect future therapy with the same or related phages (Dedrick et al. 2021; Nick et al. 2022). Furthermore, underlying immune dysregulation may persist, which may be exploited by the pathogen to evade immune surveillance (Ferreiro‐Posse et al. 2026; Liu et al. 2021; Chang et al. 2020; Wang et al. 2022).

Though phage resistance rarely evolves, it remains an ever‐present threat, particularly in cases where the smooth morphotype of *M. abscessus* is involved. Cross‐resistance may also evolve if related phages are used in the cocktail. Though the impact of host immunity on treatment outcome varies, with most treatment outcomes having little to do with immune responses, results remain exploratory due to sample size limitations and warrant larger, structured, controlled trials.

### 6.4 Challenges to Phage Therapy

Phage therapy has shown promising potential as a viable treatment alternative in the face of complex, drug‐resistant infections. However, phage therapy is faced with certain challenges. It is commonly believed that a good therapeutic candidate should be exclusively lytic, should be incapable of transduction, and should lack virulence/antibiotic resistance genes, but what actually constitutes a “good phage” remains a debated topic (Cook and Hynes 2025).

Phage therapy is a form of personalized treatment and therefore requires careful screening to identify and select a phage/cocktail that is suitable for the patient's isolate (Dedrick et al. 2019; Cristinziano et al. 2024; Little et al. 2022). This is even more challenging when the infection involves both smooth and rough morphotypes of *M. abscessus* (Cristinziano et al. 2024; Dedrick et al. 2023). While the rough morphotype of *M. abscessus* has high susceptibility to phage infection and a rare chance of resistance, the smooth morphotype is resistant (Dedrick et al. 2021). It is also worth noting that to date, all identified therapeutic candidates (Figure 3 and Table 1) are either wild type or phage derivatives of BPs, D29, Fionnbharth, Fred313, Itos, Muddy, and ZoeJ, all of which were originally isolated on *M. smegmatis* (Russell and Hatfull 2017). In fact, only a limited number of phages have been isolated on *M. abscessus* (Dedrick et al. 2019). Further efforts would therefore consider widening the repertoire of therapeutically useful phages against mycobacterial infections. Importantly, with the current understanding of mycobacterial genomics and advances in artificial intelligence, *de novo* design of viable artificial mycobacteriophages outside of natural evolution could be possible (Garyk et al. 2025; Merchant et al. 2024; Nguyen et al. 2024; King et al. 2025).

Though phage resistance rarely evolved in these case reports, the risk of phage resistance remains existent. For example, prophage‐encoded phage resistance genes are widespread in bacteria, including clinical isolates (Mageeney et al. 2020; Dedrick et al. 2017; Gentile et al. 2019; Mohammed et al. 2023). Hence, the selection of a therapeutic phage/phage cocktail ought to be meticulous and considerate of such risks so that even if resistance evolves, it may not evolve against all phages in the cocktail (Dedrick et al. 2021).

Host immunity is one of the factors believed to present a barrier to the success of phage therapy (Dedrick et al. 2023; Dedrick et al. 2021; Berkson et al. 2024; Smytheman et al. 2025). However, these case reports suggest that the impact of host immunity is variable–while antibody responses may limit the effectiveness of phage therapy in one patient (Dedrick et al. 2023; Dedrick et al. 2021; Dedrick et al. 2022), it may have minimal effect in another (Nick et al. 2022; Little et al. 2022). This suggests the likely influence of other unrecognized factors on phage therapy.

## 7 Conclusions

These case reports suggest that in the face of failed antibiotic therapy, phage therapy may offer a safe, effective alternative. Phage resistance appears to rarely evolve, and the impact of host immunity on phage therapy varies between patients, with most treatment outcomes having little to do with immune responses. However, a more pressing challenge appears to be the limited arsenal of therapeutically useful mycobacteriophages. All these three areas deserve a significant amount of attention to ensure effective and sustainable utility of mycobacteriophage therapy against drug‐resistant mycobacterial infections.

Though phage resistance appears to rarely evolve, it remains a formidable threat, especially in the case of absolute resistance (Mohammed et al. 2023). Therefore, inquiries into phage‐mycobacteria interactions would be invaluable in defining genetic factors that underlie mycobacteriophage resistance and counter‐resistance mechanisms. In addition, inquiries into phage resistance and counter‐resistance mechanisms hold a significant potential to reveal mechanisms of cell envelope assembly as well as potential phage receptors (McKitterick and Bernhardt 2022), both of which would further our understanding of phage‐host interactions and guide phage engineering efforts. Phage engineering, coevolutionary phage training/adaptation, and artificial intelligence (Dedrick et al. 2019; Borin et al. 2021; King et al. 2025) may aid efforts to expand the current limited arsenal of clinically‐useful mycobacteriophages, especially for *M. abscessus* (Dedrick et al. 2019), and particularly the smooth morphotype (Dedrick et al. 2021). It remains to be seen whether current understanding of mycobacteriophage genomics is sufficient to guide phage engineering efforts to overcome neutralizing antibody responses that may pose a serious threat to the effectiveness of mycobacteriophage therapy (Dedrick et al. 2023; Dedrick et al. 2021; Dedrick et al. 2022). This area also deserves significant research efforts.

Results from these case reports represent a great step towards achieving an effective alternative to antibiotic therapy for drug‐resistant mycobacterial infections, and it remains to be seen whether similar outcomes could be achieved for TB. Currently, phage therapy is only accessible based on compassionate use, and despite this, favorable outcomes have been recorded even in the face of complex, life‐threatening conditions. It remains to be seen whether phage therapy, alone or in combination with antibiotics, would offer similar benefits as regular therapy or during earlier stages of diseases.

## Funding

This study was supported by the National Key Research and Development Program of China (2021YFA1300904, 2023YFF0713605), Major Project of Guangzhou National Laboratory (GZNL2025C01003), and China‐Maurice Wilkins Centre (MWC) Programme Guangdong–New Zealand Bilateral Joint Funding (2025A0505020001).

## Conflicts of Interest

The authors declare no conflicts of interest.

## Data Availability

Data sharing not applicable to this article as no datasets were generated or analyzed during the current study.
